# Current practices in caesarean section training: A cross‐sectional study comparing high‐ and low‐middle‐income countries

**DOI:** 10.1002/ijgo.70696

**Published:** 2025-12-03

**Authors:** Liesl de Waard, Elize Archer, Kathryn Chu, Christoffel Joseph Brand Muller, Stefan Gebhardt

**Affiliations:** ^1^ Department of Obstetrics and Gynaecology Stellenbosch University Cape Town South Africa; ^2^ Department of Health Professions Education Stellenbosch University Cape Town South Africa; ^3^ Centre for Global Surgery Stellenbosch University Cape Town South Africa; ^4^ Department of Statistics and Actuarial Science Stellenbosch University Stellenbosch South Africa

**Keywords:** assessment, caesarean section, education, feedback, training

## Abstract

**Objective:**

This study identifies and describes global caesarean section (CS) training practices, comparing high‐income countries (HIC) and low‐ and middle‐income countries (LMIC).

**Methods:**

A convergent parallel mixed‐methods study was conducted with a cross‐sectional survey. The survey was distributed through professional networks and social media. Participation was voluntary and anonymous.

**Results:**

A total of 411 participants from 42 countries were included, with 42% (172) representing HIC and 58% (239) LMIC. Most participants were working in obstetrics and gynecology as specialists (52%, 214) or trainees (26%, 107). Participants from LMIC performed more CS annually, with a mean of 138 (±221) cases, compared to those from HIC with 44 (±64) cases (*P <* 0.001). Most were taught by an apprenticeship model (75%, 310). Feedback practices were predominantly informal, reported by 64% (263), while formal competence assessment was reported by 22% (38/172) of HIC participants and 9% (21/239) from LMIC (*P <* 0.001). Participants from LMIC completed fewer supervised cases compared to their HIC counterparts, with a median of 10 (interquartile range 5–20) compared to 50 (interquartile range 30–100) (*P <* 0.001). LMIC participants reported a higher incidence of major complications or mortality during training: 11% (24/202) versus 3% (3/120). Seventy percent (174/250) of the participants advocated for a formal training program for CS, suggesting that it could improve the quality and safety of CS.

**Conclusion:**

The study highlights current practices and differences in CS training in LMIC and HIC. The outcomes associated with CS are influenced by multiple patient‐ and system‐level factors, including access to care, patient risk profiles, and resources. However, training remains an essential and modifiable component, which, according to participants in this study, could be strengthened by incorporating evidence‐based educational practices.

## 1 INTRODUCTION

Globally, there are significant discrepancies in the clinical outcomes associated with caesarean sections (CS), with a 100‐times higher proportion of maternal deaths following CS in low‐ and middle‐income countries (LMIC) compared to high‐income countries (HIC).^1^ CS‐associated mortality is disproportionally high in LMIC, at 7.6/1000 procedures, with the highest mortality in sub‐Saharan Africa (10.9/1000 procedures).^2^ While these deaths are attributed to system and patient factors, multidisciplinary surgical training remains important in enhancing the safety and quality of CS.^2^, ^3^, ^4^

Surgical training traditionally occurs through apprenticeship, where the trainee's skill gradually develops under supervision.^5^ Advancements in evidence‐based medical education, combined with time constraints in surgery, render the apprenticeship model insufficient for surgical training.^6^ Rodriguez‐Paz (2009) proposes a new training paradigm that integrates knowledge and skills training in a simulated environment.^6^ Literature on training of CS is sparse, with available research focusing on novel programs or training of specific complications associated with CS.^7^, ^8^ Further, competency‐based training curricula for specialization programs are gaining favor over time‐based training, with structured feedback and objective structured assessment of competence built into these programs.^9^ Although direct evidence linking educational interventions to reducing maternal outcomes is limited, studies show that competency‐based and feedback‐focused training improves surgical performance, complication management, and teamwork, all of which contribute to patient safety and outcomes.^10^, ^11^ However, the extent to which these practices are implemented globally, is uncertain.

Although there are pockets of research related to the training of CS, a gap exists regarding commonly employed CS training practices and educational approaches.^7^, ^8^ Further, the existing literature does not clearly establish whether there are differences in training approaches between HIC and LMIC.^12^ This article aims to address the complexities and nuances surrounding the global teaching of CS and the educational methodologies employed.

## 2 METHODS

This was a convergent parallel mixed‐methods study, conducted using a cross‐sectional online survey. Quantitative and qualitative data were collected simultaneously, analyzed separately, and integrated during interpretation. This approach was chosen to capture frequencies from quantitative data and deeper insights into these from open‐ended qualitative questions.^13^, ^14^ The primary outcome was to determine teaching and supervision practices and compare these between HIC and LMIC, based on World Bank income designation.^12^

The survey was conducted from October 2023 to January 2024 and constructed using REDCap software.^15^ A link to the survey was distributed through email and on an electronic pamphlet with a QR code. Invitations were distributed to the members of the South African Society of Obstetrics and Gynecology (SASOG) and other international Obstetrics and Gynecology (O&G) groups. The primary investigator distributed the pamphlet in person at the FIGO (International Federation of Gynecology and Obstetrics) conference in Paris, at the SASOG booth, and at a meeting of the World Association of Trainees in Obstetrics and Gynecology. A snowball sampling approach was used, where interested individuals were asked to disseminate the link to their colleagues.^16^ Surveys were completed independently and anonymously on participants' own devices. The target population included doctors involved in performing or teaching CS. The inclusion criteria were any medical doctors who have performed CS. The exclusion criteria were non‐physician practitioners or medical doctors who have not performed CS in their careers.^12^

### 2.1 Survey tool

The survey was formulated by authors (LW, SG) following a comprehensive review of the literature on clinical and surgical skills training. Educational theories considered included the three‐stage motor skill acquisition model of Fits and Posner and Kolb's learning cycle.^17^, ^18^ A pragmatic mixed‐methods approach was used, combining multiple‐choice, Likert‐scale, and open‐ended questions to gather real‐world data.^19^ The survey tool underwent pilot testing with a cohort (*n* = 10) from the Department of O&G at Tygerberg Hospital, Cape Town. Subsequently, certain questions were refined to enhance clarity. The final survey tool comprised 38 questions, categorized into five domains (Data S1):

1. Participant demographics.
2. CS performance experience.
3. CS training experience.
4. Self‐assessment of CS personal training.
5. CS teaching strategies.

### 2.2 Consent

Participation in the study was anonymous and voluntary, and the survey's first question obtained informed consent from participants. The research proposal was approved by the Health Research and Ethics Committee of Stellenbosch University (S22/11/263). All questions were optional, allowing participants to answer only those relevant to them. To draw attention to the survey, a lucky draw of US$100 was offered for which the participants could provide their email address at the end of the survey. These email addresses were stored separately and not associated with their survey responses.

### 2.3 Analysis

Upon completion, the data were extracted into Microsoft Excel. For the qualitative component, descriptive statistics, including frequencies, means with standard deviation, and medians with interquartile ranges, were calculated based on the number of respondents who answered each question. Results of questions were compared based between HIC and LMIC of the country where the participants were trained to perform CS.^12^ Numerical variables were compared using appropriate *t*‐tests for means after assessing the equality of variances using an *F*‐test and Wilcoxon–Mann–Whitney tests. Categorical variables were compared using *χ* ^2^‐tests of association and Fisher's exact test. Statistical data analysis was conducted using SAS software, version 9.4 of the SAS System for Windows (SAS Institute). Missing data was handled by calculating proportions for each variable based on the number of participants who completed that specific question. The data was screened manually for possible duplicate entries.

The qualitative component consisted of four open‐ended questions embedded within the survey. Responses were analyzed using content analysis principles to identify recurring themes and patterns.^20^ The quantitative data provided details on scope and prevalence, and the qualitative data improved the context and understanding and were integrated into the discussion section.

## 3 RESULTS

**FIGURE 1:**
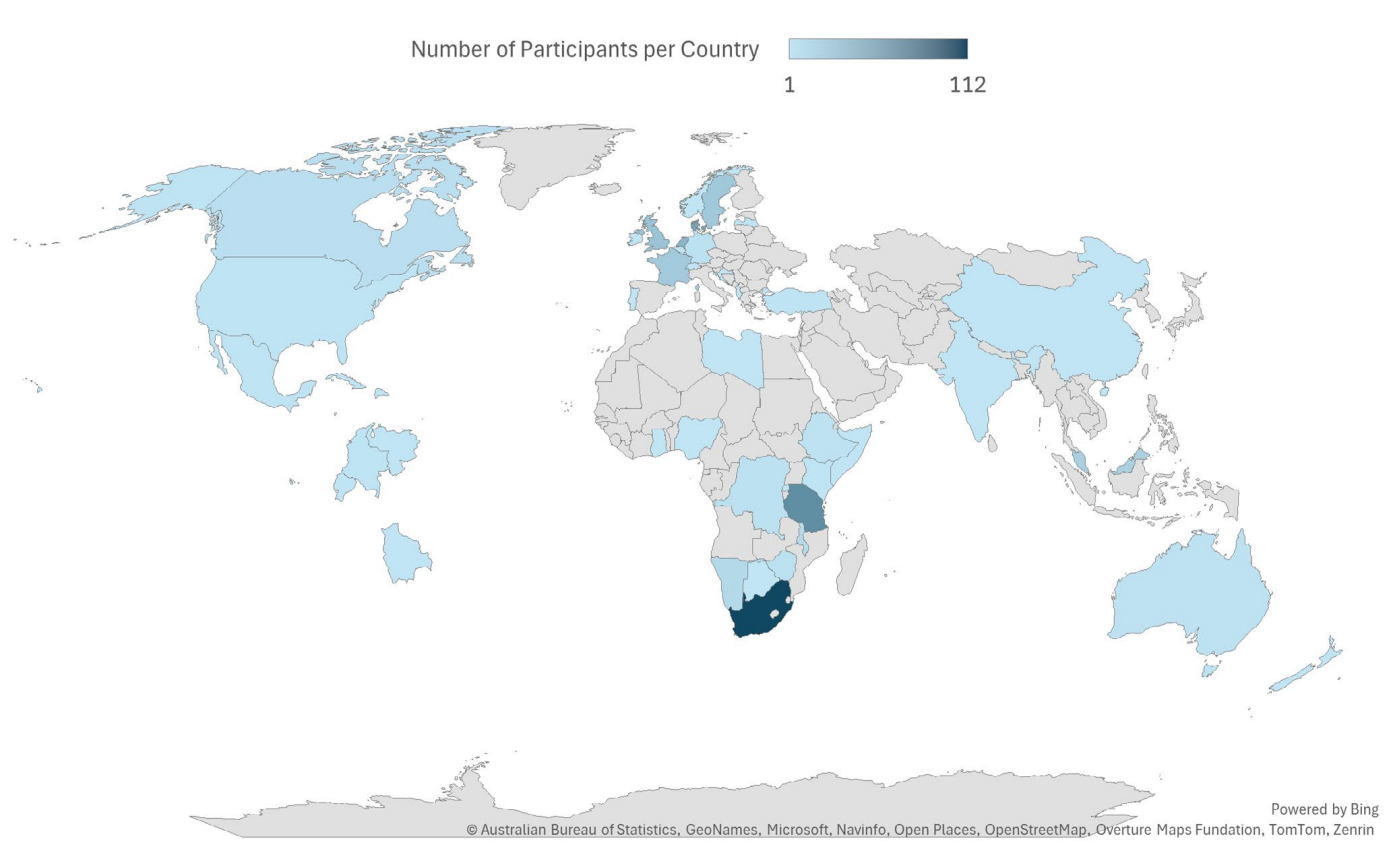
Distribution of participants by country of caesarean section training.

The survey was completed by 411 participants from 42 countries, with 172 (42%) trained in HIC and 239 (58%) in LMIC. Figure 1 illustrates the global distribution of participants, and Table 1 provides a breakdown of participants according to their respective training locations for CS.

**TABLE 1:** Participants and their respective training locations where they perform caesarean sections.

Country where participants trained to perform caesarean sections	Number of participants per country listed *N* (%)
South Africa	112 (27)
Tanzania	62 (15)
Denmark	47 (11)
Netherlands	34 (8)
United Kingdom	24 (6)
Sweden	21 (5)
France	20 (5)
Malaysia	15 (4)
Namibia	9 (2)
Malawi	8 (2)
Canada	5 (1)
Democratic Republic of the Congo, Germany, Zimbabwe, (four participants per country)	12 (3)
Australia, Ethiopia (three participants per country)	6 (2)
Belgium, Colombia, Kenya, Latvia, Mexico, Turkey, United States (two participants per country)	14 (3)
Albania, Barbados, Bolivia, Botswana, China, Croatia, Cuba, Dominican Republic, Ecuador, Ghana, India, Libya, Mauritius, New Zealand, Nigeria, Norway, Portugal, Republic of Ireland, Rwanda, Somalia, Switzerland, Venezuela (one participant per country)	22 (5)

The demographic characteristics and background related to CS performance of the participants are listed in Table 2. The mean age of the cohort was 40 (±11) years, with a predominance of female participants, 282 (69%). Most participants were specialists in O&G, 214 (53%), and 107 (26%) were trainees in this field.

**TABLE 2:** Demographic, employment and caesarean section training background: Comparing high income country and low middle‐income country participants.

Survey question	All	HIC	LMIC	*P*‐value
What is your age? (*N*)^a^	411	172	239	
Mean [±SD]	40 [11]	38 [10]	42 [11]	<0.001
Which gender do you identify with?				
Female (%)	282 (69)	149 (87)	133 (56)	<0.001
Male (%)	129 (31)	23 (13)	106 (44)	
What year did you complete your pre‐graduate medical training (your primary medical degree)? Med [IQR]	2011 [2004–2016]	2014 [2006–2018]	2009 [2003–2014]	<0.001
In the Country you trained in, what work do you do immediately after your primary medical degree? (*N*)	408 (%)	171 (%)	237 (%)	
1. In your first post‐graduate year, you have an internship (work supervised in different disciplines)	275 (67)	89 (52)	186 (78)	<0.001
2. Work supervised as an intern in a chosen discipline	82 (20)	49 (29)	33 (14)	
3. You start specializing immediately after your medical degree	36 (9)	27 (16)	9 (4)	
4. Other	15 (4)	6 (4)	9 (4)	
What is your current job title? (*N*)	409 (%)	172 (%)	237 (%)	
1. Intern (year‐one post‐graduate training, not specializing, working under supervision)	12 (3)	6 (3)	6 (3)	<0.001
2. Medical officer (post‐graduate non‐specialist doctor)	31 (8)	9 (5)	22 (9)	
3. Registrar/trainee specializing in another discipline	10 (2)	8 (5)	2 (1)	
4. Registrar/trainee specializing in obstetrics and gynecology	107 (26)	70 (41)	37 (16)	
5. Specialist, obstetrics and gynecology	214 (52)	64 (37)	150 (63)	
6. Specialist, other discipline	27 (7)	11 (6)	16 (7)	
7. Other	8 (2)	4 (2)	4 (2)	
How many years have you worked in an obstetric service where you are or were involved with performing CS? (*N*)	410 (%)	172 (%)	238 (%)	
1. 0–10	249 (61)	120 (70)	129 (54)	0.005
2. 11–20 years	105 (26)	36 (21)	69 (29)	
3. >20	56 (14)	16 (9)	40 (17)	
Are you currently performing CS?	394 (%)	164 (%)	230 (%)	
1. Yes, independently	305 (77)	111 (68)	194 (84)	<0.001
2. Yes, under supervision	56 (14)	45 (27)	11 (5)	
3. No, I am in early training	5 (1)	3 (2)	2 (1)	
4. Other^b^	28 (7)	5 (3)	23 (10)	

Abbreviations: CS, caesarean section; HIC, high‐income countries; IQR, interquartile range; LMIC, low‐ and middle‐income countries; SD, standard deviation.

^a^ *N* = number of participants that answered the question.

^b^ Previously performed CS but not currently due to retirement (2), working in other specialities or fields (26).

### 3.1 The training process

Participants trained in LMIC performed a significantly higher total number of CS, with a mean of 138 (±221) CS performed annually in LMIC, compared to 44 (±64) in HIC. Clinicians who mostly performed CS in the participants' countries of training were O&G specialists, 92 (54%) in HIC, and non‐specialist doctors,130 (55%) in LMIC. Most participants (310, 75%) received CS training by apprenticeship. Feedback during training was predominantly informal, occurring during the procedure (263, 64%). Formal and written feedback was reported to be significantly more common among participants from HIC. Questions related to the training process are captured in Table 3.

**TABLE 3:** Overview and comparison of the caesarean section training process by country's income stratification.

Survey question	ALL	HIC	LMIC	*P*‐value
In the country that you are working in, who primarily performs caesarean sections? *N* ^a^	409 (%)	171 (%)	238 (%)	
1. Doctors in training as specialists in obstetrics and gynecology	95 (23)	62 (36)	33 (14)	<0.001
2. Specialist in obstetrics and gynecology	150 (37)	92 (54)	58 (24)	
3. Non‐specialist doctors who have completed their training	141 (34)	11 (6)	130 (55)	
4. Other	21 (5)	6 (4)	15 (6)	
5. Other medical specialists	2 (0.5)	0 (0)	2 (1)	
What year did you start performing caesarean sections? Median [IQR]	2013 [2006–2018]	2014 [2006–2018]	2011 [2005–2016]	<0.001
Approximately how many caesarean sections have you performed independently? Mean [±SD]	1577 [3880]	641 [1422]	2233 [4815]	<0.001
Approximate number of caesarean sections performed per year.^b^ Mean [±SD]	99 [180]	44 [64]	138 [221]	<0.001
How many years after completing your pre‐graduate medical degree did you start with caesarean section training? Med (IQR)	*n* = 353 1 [1–2]	*n* = 140 2 [1–3]	*n* = 213 1 [1–2]	<0.001
Did you receive any training for caesarean sections during your primary medical degree? (select all that apply) (*N*)	411 (%)	172 (%)	239 (%)	
1. No	75 (18)	47 (27)	28 (12)	<0.001
2. Yes, lectures only	59 (14)	21 (12)	38 (16)	0.321
3. Yes, observation only	114 (28)	48 (28)	66 (28)	1.000
4. Yes, skills simulation/in skills lab	11 (3)	1 (1)	10 (4)	0.029
5. Yes, in the operating room assisting	161 (39)	51 (29)	110 (46)	0.001
6. Yes, in the operating room performing certain aspects (e.g., closing the rectus sheath)	58 (14)	21 (12)	37 (15)	0.390
7. Yes, in the operating room performing the complete procedure	31 (8)	11 (6)	20 (8)	0.571
Regarding your training in caesarean sections, was there a formal curriculum/training program used for the caesarean section training? (choose all that apply) (*N*)	411 (%)	172 (%)	239 (%)	
1. No	215 (52)	94 (55)	121 (51)	0.425
2. Yes, during my primary medical degree	37 (9)	4 (2)	33 (14)	<0.001
3. Yes, after my primary medical degree	45 (11)	6 (3)	39 (16)	<0.001
4. Yes, during my speciality training	78 (19)	34 (20)	44 (18)	0.799
Which of the following methods were used for your caesarean section training? (select all that apply) (*N*)	411 (%)	172 (%)	239 (%)	
1. Apprenticeship model (assist until trainer deems you competent to perform the caesarean section)	310 (75)	129 (75)	181 (76)	0.908
2. Simulation training (simulators/models used to teach steps of a caesarean section)	19 (5)	6 (3)	13 (5)	0.477
3. Pre‐surgical training courses/modules	49 (12)	19 (11)	30 (13)	0.758
4. Observation only	55 (13)	13 (8)	42 (18)	0.003
How was feedback given during training? (select all that apply) (*N*)	411 (%)	172 (%)	239 (%)	
1. Written	53 (13)	42 (24)	11 (5)	<0.001
2. Formal verbal feedback (i.e., trainer and trainee have a planned discussion regarding the details of the surgical progress)	123 (30)	78 (45)	45 (20)	<0.001
3. Informal verbal feedback during and after cases (comments on technique and progress)	263 (64)	126 (73)	137 (57)	<0.001
4. Correcting techniques or steps during the procedure, for the sake of patient safety	254 (62)	118 (69)	136 (57)	0.018
5. None/I am not aware of any feedback being given	12 (3)	0 (0)	12 (5)	0.002

Abbreviations: HIC, high‐income countries; IQR, interquartile range; LMIC, low‐ and middle‐income countries; SD, standard deviation.

^a^ *N* = number of participants who answered the question.

^b^ Calculated from the number of years since working and the number of estimated cases performed.

Details about the participants' experiences of the training process are outlined in Table 4. Participants assisted in a comparable duration and number of CS procedures before beginning to perform parts of the procedure. Participants from HIC performed more partial cases and significantly more supervised cases before performing CS independently. Regarding the assessment of competence, 131 participants (32%) indicated that there was no formal evaluation process in place. The practice of formal competence assessments was more prevalent among participants trained in HIC, with 38 (22%) participants reporting it, compared to 21 (9%) in LMIC (*P* < 0.001).

**TABLE 4:** Participants' experiences with the caesarean section training process.

Survey question	ALL	HIC	LMIC	*P*‐value
Approximately how many cases did you assist with before you started performing parts of a caesarean section on your own? Med [IQR]	*N* ^a^ = 350 10 [5–25]	*N* = 137 10 [5–20]	*N* = 213 15 [10–30]	0.030
Approximately how long did you assist before you started performing parts of a caesarean section on your own? (*N*)	354 (%)	140 (%)	214 (%)	
1. <1 month	155 (44%)	58 (41)	97 (44)	0.886
2. 1–6 months	148 (42)	61 (44)	87 (41)	
3. 0.5–1 year	33 (9)	13 (9)	20 (9)	
4. 1–5 years	18 (5)	8 (6)	10 (5)	
Approximately how many cases did you perform partially before your first entire caesarean section? Med [IQR]	*n* = 347 10 [5–20]	*n =* 136 10 [5–20]	*n =* 211 6 [4–15]	0.001
Approximately how many cases did you perform supervised before you performed caesarean sections independently? Med [IQR]	*N* = 337 15 [10–50]	*N* = 128 50 [30–100]	*N* = 209 10 [5–20]	<0.001
At what stage in your career did you perform caesarean sections independently? (assisted by someone at a lower level of experience than yourself) (*N*)	347 (%)	133 (%)	214 (%)	
1. Intern (year one post‐graduate training, not specializing, working under supervision)	74 (21)	11 (8)	63 (29)	<0.001
2. Medical officer (post‐graduate but non‐specialist doctor)	128 (37)	10 (8)	118 (55)	
3. Registrar/trainee specializing in another discipline	10 (3)	10 (8)	0 (0)	
4. Registrar/trainee specializing in obstetrics and gynecology	107 (31)	87 (65)	20 (9)	
5. Specialist, obstetrics and gynecology	10 (3)	8 (6)	2 (1)	
6. Specialist, other discipline	1 (0.5)	1 (1)	0 (0)	
7. Other	17 (5)	6 (5)	12 (5)	
Was there any formal process to determine your competence prior to you performing a caesarean section independently? (select all that apply) (*N*)	411 (%)	172 (%)	239 (%)	
1. No, there was no formal process to determine my competence	131 (32)	36 (21)	95 (40)	<0.001
2. Yes, informal assessment by supervisors	156 (38)	63 (37)	93 (39)	0.681
3. Yes, formal structured assessment by supervisor	59 (14)	38 (22)	21 (9)	<0.001
4. Yes, logbook or portfolio assessment	72 (18)	42 (24)	30 (13)	<0.001

Abbreviations: HIC, high‐income countries; IQR, interquartile range; LMIC, low‐ and middle‐income countries; SD, standard deviation.

^a^ *N* = number of participants who answered the question.

### 3.2 Experience of training

**FIGURE 2:**
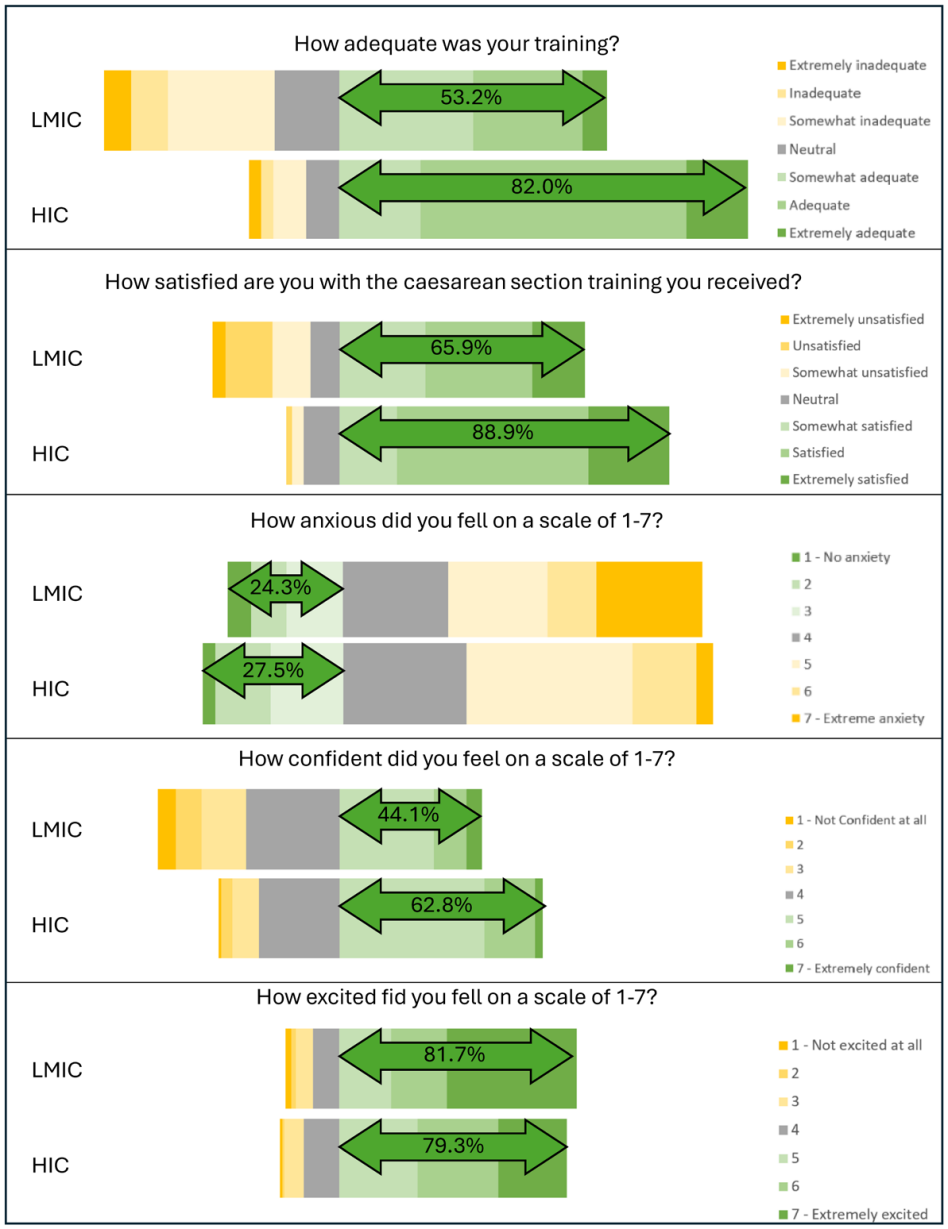
Participants' experience of the training process.

The participants' rating of their training experience was evaluated using five questions, each scored on a seven‐point Likert scale (Figure 2 and Table 5). Participants from LMIC rated their training as less adequate compared to those from HIC (*P* < 0.001). They also reported being more anxious when performing their first CS (*P* < 0.001). Overall, the participants from HIC were significantly more satisfied with their training than those from LMIC (*P* < 0.001). Participants from LMIC reported more major complications during their first year of performing CS, with 21 (10%) cases compared to only one (0.83%) case from HIC.

**TABLE 5:** Comparison of the rating of the training experiences.

Survey question	ALL	HIC	LMIC	*P*‐value
When you performed your first caesarean section independently, reflecting back, how adequate was your training? Mean 1–7 [±SD]	4.73 [1.58]	5.41 [1.35]	4.33 [1.57]	<0.001
*N* ^a^	325 (%)	122 (%)	203 (%)	
1. Extremely inadequate	14 (4)	3 (4)	11 (5)	<0.001
2. Inadequate	18 (6)	3 (4)	15 (7)	
3. Somewhat inadequate	51 (16)	8 (7)	43 (21)	
4. Neutral	34 (10)	8 (7)	26 (12)	
5. Somewhat adequate	74 (23)	20 (16)	54 (26)	
6. Adequate	109 (34)	65 (53)	44 (21)	
7. Extremely adequate	25 (8)	15 (12)	10 (4)	
When you performed your first caesarean section independently, how anxious did you feel on a scale of 1–7? Mean [±SD]	4.51 [1.63]	4.24 [1.37]	4.67 [1.75]	0.016
*N*	322 (%)	120 (%)	202 (%)	
1. Not anxious at all	13 (4)	3 (3)	10 (5)	<0.001
2.	28 (9)	13 (11)	15 (7)	
3.	41 (13)	17 (14)	24 (12)	
4.	74 (23)	29 (24)	45 (22)	
5.	81 (25)	39 (33)	42 (21)	
6.	36 (11)	15 (3)	21 (10)	
7. Extremely anxious	49 (15)	4 (3)	45 (22)	
When you performed your first caesarean section independently, how confident did you feel on a scale of 1–7? Mean [±SD]	4.36 [1.33]	4.66 [1.08]	4.18 [1.43]	<0.001
*N*	323 (%)	121 (%)	202 (%)	
1. Not confident at all	12 (4)	1 (1)	11 (5)	0.007
2.	20 (6)	4 (3)	16 (8)	
3.	38 (12)	10 (8)	28 (14)	
4.	88 (27)	30 (25)	58 (29)	
5.	113 (35)	54 (45)	59 (29)	
6.	39 (12)	19 (16)	20 (10)	
7. Extremely confident	13 (4)	3 (2)	10 (5)	
When you performed your first caesarean section independently, how excited did you feel on a scale of 1–7? Mean [±SD]	5.64 [1.42]	5.45 [1.28]	5.75 [1.48]	0.0601
*N*	323 (%)	121 (%)	202 (%)	
1. Not excited at all	5 (2)	1 (1)	4 (2)	0.164
2.	4 (1)	1 (1)	3 (1)	
3.	20 (6)	8 (7)	12 (6)	
4.	33 (10)	15 (12)	18 (9)	
5.	69 (21)	33 (27)	36 (18)	
6.	73 (23)	34 (28)	39 (19)	
7. Extremely excited	119 (37)	29 (24)	90 (45)	
How satisfied are you with the caesarean section training you received? Mean [±SD]	5.14 [1.58]	5.72 [1.09]	4.77 [1.73]	<0.001
*N*	333 (%)	128 (%)	205 (%)	
1. Extremely unsatisfied	7 (2)	0 (0)	7 (3)	<0.001
2. Unsatisfied	28 (8)	2 (2)	26 (13)	
3. Somewhat unsatisfied	25 (8)	4 (3)	21 (10)	
4. Neutral	28 (8)	12 (9)	16 (8)	
5. Somewhat satisfied	66 (18)	19 (15)	47 (23)	
6. Satisfied	123 (37)	64 (50)	59 (29)	
7. Extremely satisfied	56 (17)	27 (21)	29 (14)	
Did you experience complications during your first year of performing caesarean sections independently? (*N*)				
*N*	322 (%)	120 (%)	202 (%)	
1. No, I did not experience complications that I am aware of	149 (46)	69 (58)	80 (40)	0.001
2. Yes, I experienced minor surgical complications requiring correction by a senior colleague during the procedure	144 (45)	48 (40)	96 (48)	
3. Yes, I experienced major surgical complications requiring additional surgical procedures such as a relook laparotomy	22 (7)	1 (1)	21 (10)	
4. Yes, I experienced surgical complications and surgical‐associated maternal mortality	5 (1)	2 (2)	3 (1)	
5. Yes, I experienced surgical‐associated maternal mortality	2 (1)	0 (0)	2 (1)	

Abbreviations: HIC, high‐income countries; IQR, interquartile range; LMIC, low‐ and middle‐income countries; SD, standard deviation.

^a^ *N* = number of participants who answered the.

### 3.3 Caesarean section teaching practices

Question responses on how participants train others are shown in Table 6. Apprenticeship remains the most common method, with an increasing use of simulation and pre‐surgical training. Formal feedback is widely utilized, and more so in HIC.

**TABLE 6:** Questions on caesarean section teaching practices by country category.

Survey question	ALL	HIC	LMIC	*P*‐value
Are you currently training others to perform caesarean section? *N* ^a^ (%) Yes	190 (46)	74 (43)	116 (49)	
Do you use any formal training program/curriculum while training others? *N* (%) Yes	54 (29)	23 (31)	31 (27)	0.645
Which of the following methods do you use for caesarean section training? (select all that apply) (*N*)	190 (%)	74 (%)	116 (%)	
1. Apprenticeship model (assist until trainer deems you competent to perform the caesarean section) (Select all that apply)	167 (88)	71 (49)	96 (83)	0.006
2. Simulation training—simulators/models used to teach steps of caesarean section	34 (18)	10 (14)	24 (21)	0.247
3. Pre‐surgical training (e.g., lectures, tutorials, online content)	57 (30)	12 (16)	45 (39)	0.001
i. Lectures	36 (19)	7 (9)	29 (25)	0.008
ii. Tutorials	35 (18)	5 (7)	30 (26)	<0.001
iii. Online content	33 (17)	8 (11)	25 (22)	0.076
4. Observation only	28 (15)	10 (14)	18 (16)	0.835
How is feedback given during the caesarean section training you provide? (select all that apply) *N*	190 (%)	74 (%)	116 (%)	
1. Written	30 (16)	22 (30)	8 (7)	<0.001
2. Formal verbal feedback (i.e., the trainer and trainee have a planned discussion regarding the details of the surgical progress)	85 (45)	42 (57)	43 (37)	0.011
3. Informal verbal feedback during and after cases (comments on technique and progress)	148 (78)	68 (92)	80 (7)	<0.001
4. Correcting techniques or steps during the procedure, for the sake of patient safety	128 (68)	62 (84)	66 (57)	<0.001
5. None/I am unaware of feedback be	2 (1)	0 (0)	2 (2)	0.522
Do you think there is a need for a formal training program for caesarean sections? *N*	250 (%)	91 (%)	159 (%)	
1. No	31 (12)	25 (27)	6 (4)	<0.001
2. Unsure	45 (18)	28 (31)	17 (11)	
3. Yes	174 (70)	38 (42)	136 (86)	

Abbreviations: LMIC, low‐ and middle‐income countries; HIC, high‐income countries.

^a^ *N* = number of participants who answered the question.

When participants were asked if they believed there was a need for a formal training program in CS, 174 (69.6%) believed such a program is necessary, with stronger support among participants from LMIC.

### 3.4 Open‐ended questions

The survey included four open‐ended questions focused on how participants thought CS training could be improved:

1. How do you think CS training could be improved?
2. What do you think is good about CS training as it is now?
3. You indicated you believe there is a need for a formal training program for CS. What kind of program would you suggest?
4. What would be the expected benefits of having a formal training program for CS?

The themes identified from the data are summarized below with example quotes for themes presented in Tables 7 and 8.

**TABLE 7:** Themes and example quotes of open‐ended questions: Question 1 and 3.

Question 1: How do you think caesarean section training could be improved?	
Question 3: You indicated you believe there is a need for a formal training program for caesarean sections. What kind of program would you suggest	
The need for a formal curriculum	“Formal training. Video tutorial teaching on potential complications and how to manage them, formal assessment of skills” “Formal program beforehand (cadaver/simulation training), formal explanation beforehand/lecture” “It needs more formality as the apprenticeship model has been eroded. As a senior I don't see the same trainee operate to teach them” “Lectures on anatomy relating to a caesarean section, the procedure itself aided by a video. A practical session on how to hold surgical instruments then simulation on how to perform a caesarean section. Then move to observation and assisting the procedure. From then on verbal feedback during procedure with formal assessment at an interval.” “Structured curriculum Complications workshop Formal feedback” “Module on safe surgical techniques during medical school. Sim training. Observation Feedback during and after procedure Logbook Most of all: Being a role model for safe surgery with sound and safe techniques employed at all times.”
Required knowledge and skills	“Understanding anatomy more” “To have more lectures on complications and complicated c sections” “Practical lecture on the anatomy and physiology of the uterus, ureter, bladder and other adjacent organs. Understanding natural birth. Understanding the indications. Do things with knowledge” “Importantly first by standardizing the procedure of caesarean section and then start training knowledge then use of manikins emphasizing on steps and precautions needed to be taken, then to actual assisting and get formal feedback from the supervisor who is senior and has correct steps of doing caesarean section.” “Having longer and more comprehensive simulation and theater sessions from mid medical school” “Increasing training with simulation environments during undergraduate training… and during the postgraduate period as general practitioners” “Know the anatomy Simulation Observe Assist” “Module on safe surgical techniques during medical school. Sim training. Observation Feedback during and after procedure Logbook”
The need for mentorship and supervision	**“**I only ever cut one Caesar with a consultant in my entire medical officer, comm serve, internship and registrar training. I was only ever trained by other medical officers and other registrars” “Cut more under supervision of consultants.” “Direct supervision until a trainee is competent enough” “Consistent, focused high‐volume exposure assisting senior consultants early” There were suggestions from participants from HIC for earlier independent surgery. “Very long supervised period before performing the procedure independently” “Earlier Independent as you truly start learning when along operating. I also worked in (LMIC), where colleagues did far less procedures before operating below. An optimum lies in the middle, but 50–60 with supervision is not necessary” “Module on safe surgical techniques during medical school. Sim training. Observation Feedback during and after procedure Logbook Most of all: Being a role model for safe surgery with sound and safe techniques employed at all times.” “Definitely a knowledge test to see if someone is fully up to speed with recent evidence base practices. maybe simulator training, but this is not feasible in district hospitals maybe. Maybe a teach the teacher program for the supervisors”
Feedback and formal assessment	“There should be a formal performance evaluation for safe c‐section prior to be given a go ahead to perform independently. This should involve a minimum requirement to perform under supervision and later minimal requirement to perform independently so that we minimize surgical complications that have been on the rise recently.” “The current system in the UK is a slow apprenticeship model gradually increasing complexity over many years. The system is very safe but relies on high levels of specialist staffing 24/7” “Formal teaching, apprenticeship, formal assessment” “Have a structured formal system of stepwise training with formal assessment of competence” “More formal assessment before doing CS independently. Peer training‐ I learned the most from assisting my fellow registrars.” “Theory testing Practical skill testing with models Structured assessments” “Tiered approach starting in medical school. Lectures, theory, videos, simulation, assisting an experienced surgeon who consciously verbalizes the process, step‐wise approach to doing parts of the caesarean with feedback after each session.” “Various types of CS to be signed off once the supervisor is satisfied the trainee can perform independently and safely. Also, more comprehensive program regarding decision‐making and care before, during and after CS”

**TABLE 8:** Themes and example quotes of open‐ended questions: Question 2 and 4.

Question 2: What do you think is good about caesarean section training as it is now?	
Exposure and early independence	“Being thrown in the deep side makes you learn to swim faster”
	“Allows for practice due to number of cases and increase in personnel confidence as you do more.”
	“Being independent as soon as possible”
	“Currently it gives the young doctors exposure at an earlier stage in their careers. It falls under the category of “necessity breeds skill” one of the things in life I learned to do because no one else could do it and I am somewhat proud of that accomplishment!”
	“Very long supervised period before performing the procedure independently”
Supervision and stepwise training	“The current system in the UK is a slow apprenticeship model gradually increasing complexity over many years. The system is very safe but relies on high levels of specialist staffing 24/7”
	“Lot of exposure, starting early to observe and assist during CS, step by step approach to become more independent”
	“Lots of volume with supervision to get lots of hands on experience and real‐time feedback.”

Question 4: What do you believe would be the expected benefits of having a formal training program for caesarean sections?	
Improve equality of CS globally	“All residents are trained in a more equal way.”
	“The skill and quality at a certain level of experience would be uniform throughout the country.”
	“Ensuring patient safety. Equitable skills”
	“Same standard of care and not depended on the supervisor you work with, especially in LMIC bad habits are taught to new doctors over and over again.”
	“Worldwide protocols and standards facilitate monitoring and evaluation and reference for research purposes”
Improve patient outcomes	“Decrease in surgically associated mortality and morbidity”
	“Improved patient care and outcomes”
	“Less complications, fewer instances of the supervisor taking over, less bleeding, trainee feeling more confident and better self‐worth.”
	“Less complications associated with caesarean sections. Less bleeding. Less morbidity and mortality.”
Improve the experience of the trainee	“Less trauma to both the surgeons who are still learning and the patients (complications) It would inspire colleagues to set up formal training programs in smaller/more remote areas that they may then go and work at. Avoid wrong habits that may have a negative impact on a high number of patients (both surgical techniques and communication skills)”
	“Safer C/S Decreased maternal mortality and morbidity Less anxiety under staff involved”
	“More confidence earlier in training”

#### 3.4.1 Questions 1 and 3

When analyzing the content of these two questions, similar themes arose for both, and they were grouped together (Table 7).

##### 3.4.1.1 The need for a formal curriculum

Participants advocated for the formalization and structuring of training. They proposed a scaffolded approach, beginning with basic knowledge and skills, progressing through simulation and supervised practice, and culminating in assessment.

##### 3.4.1.2 Required knowledge and skills

Many suggested incorporating formal content, including anatomy, surgical skills training, and simulation, into CS training. Training that focuses on managing complications was highlighted as important and a standardized approach to performing CS. Many participants from LMIC proposed that CS training should be included in undergraduate degrees.

##### 3.4.1.3 The need for mentorship and supervision

Several participants, particularly those from LMIC, recommended increased supervision by specialists or seniors and importance of supervisor continuity. There were further suggestions on role‐modeling and providing training for trainers.

##### 3.4.1.4 Feedback and formal assessment

Participants from both HIC and LMIC highlighted the importance of incorporating feedback and competence assessments into training. They emphasized that this process should be conducted in a respectful and supportive manner, avoiding degradation.

#### 3.4.2 Question 2

For this question, two themes emerged (Table 8).

##### 3.4.2.1 Exposure and early independence

The majority of participants from LMIC highlighted the value of exposure and early independence.

##### 3.4.2.2 Supervision and stepwise training

Participants from HIC primarily highlighted the benefits of supervision, stepwise training, and slow apprenticeship, with competency assessment and feedback.

#### 3.4.3 Question 4

Three key themes emerged from this question (Table 8).

##### 3.4.3.1 Improve equality of CS globally

Several participants suggested that a formal program would improve the equality of how doctors are trained and that the training quality would not depend on supervisor motivation.

##### 3.4.3.2 Improve patient outcomes

This theme encompassed most suggestions for this question, focusing on how a formal training program could improve patient outcomes. Potential improvements mentioned were decreasing morbidity and mortality and decreasing blood loss.

##### 3.4.3.3 Improve the experience of the trainee

The third theme emphasized optimizing the trainee's experience of training. These suggestions were often related to patient complications that caused trauma and anxiety, as well as improving the confidence of the trainee.

The suggestion from the open‐ended questions highlighted participants' training experiences and echoed the survey findings, particularly regarding the emphasis on independence and limited supervision in LMIC.

## 4 DISCUSSION

This survey analyzed the experiences and perspectives of 411 participants who received clinical training in 42 countries, encompassing all continents. The findings revealed notable differences in surgical training practices between HIC and LMIC.

This survey showed that doctors in LMIC commence independent surgical practice at an earlier stage and perform a greater number of CS with procedures predominantly carried out by non‐specialist doctors. In contrast, in HIC, surgical training typically begins within specialized training programs, incorporating a more gradual and supervised learning process. These differences in experiences of training and supervising are not surprising considering the differences in physician‐to‐population ratios, estimated at 3.6/1000 in HIC versus 1.3/1000 in LMIC.^21^

Participants from LMIC reported a higher incidence of major complications and mortality associated with their surgical training compared to those from HIC. These findings align with data indicating CS‐associated maternal mortality of a hundred times higher in LMIC than in HIC.^2^ Quantifying the impact of CS training practices on mortality rates is challenging, as multiple clinical, resource‐related, and social determinants of health contribute to overall outcomes.^2^ Of concern is the limited supervision reported by participants from LMIC, considering the learning curve for CS, in relation to operative time and blood loss, typically stabilizes after 15–50 procedures.^22^ Inadequate supervision might contribute to poor outcomes and dismissing the role of training practices in LMIC as a possible contributing factor would be inappropriate. Maternal morbidity and mortality not only represent an enormous societal burden but also place immense strain on doctors, who must navigate complex situations.^23^

Most participants reported being trained primarily through apprenticeship, with limited exposure to simulation‐based training or pre‐surgical training courses. These trends did not differ based on country income designation. Simulation training in CS teaching has been well documented as beneficial, particularly for developing proficiency in managing complications and enhancing teamwork and communication, both of which have been associated with improved clinical outcomes.^24^ Expanding access to these training methods should be considered in both HIC and LMIC.

A significant difference was observed in the implementation of feedback and competency assessment within training programs. The integration of feedback in clinical skills training has been recognized as a crucial element of Kolb's learning cycle.^18^ Improving the effectiveness of feedback and fostering a culture of feedback is complex. However, employing a systematic approach has demonstrated positive educational outcomes.^25^ Evidence supports the adoption of competency‐based rather than time‐based training models; specific competency assessments have been developed for CS training.^10^, ^11^ The described tools were reported to have learning curves necessitating adequate training for instructors to ensure their optimal use.^10^ These tools have been successfully implemented in some LMIC.^11^

Many participants recommended establishing a formal training program for CS. Proposed elements include pre‐surgical skills and knowledge training, standardization of the procedure and competency assessment with enhanced supervision. When evaluating a standardized approach to CS, various techniques have been debated and evidence‐based CS techniques described.^26^ However, factors such as individual preferences, the influence of mentors and senior opinions, availability of resources, and varying skill levels all play a role in determining surgical practices.^27^ FIGO has published comprehensive guidelines outlining best practices for CS surgical techniques.^28^ This guideline can be used as a standard for teaching and training.

High‐income country recommendations emphasize earlier opportunities for independent surgical practice and increased procedural volume. Proposed solutions include global surgical exchange programs, although some raise concerns about the ethical implications.^29^ Ng‐Kamstra (2025) have provides guidance on ethical approaches to global surgical rotations, where HICs collaborate with LMICs for responsible engagement and sustainable collaboration.^29^

### 4.1 Strengths

The strength of the study lies in global representation, capturing a range of training practices across different regions. To our knowledge, this is the first study to explore CS training in this way. The survey featured a variety of questions, including open‐ended options, allowing participants to provide insights beyond the predefined choices. Most respondents were specialists or trainees in O&G, ensuring strong representation from experts in the field.

### 4.2 Limitations

Surveys as a research method have well‐recognized limitations, including participation bias. Because most respondents were trainees and specialists in O&G, the perspectives might be skewed toward those with a positive experience and a strong interest in CS training. Difference in participants' professional designation likely influenced their training and might limit conclusions from direct comparisons. The survey was conducted in English only, which presents a notable limitation. As the snowball approach was used, the precise number of individuals who were offered the survey cannot be determined.

Further research is necessary to investigate training quality and access, given the growing concern about inequalities in global maternal mortality.^30^ Collaboration between HIC and LMIC could offer a potential solution, helping to balance service delivery demands with the need for a more comprehensive training approach. In terms of patient‐centeredness and hearing the voices of patients, perspectives of patients undergoing CS procedures, particularly those performed by junior trainees, must also be actively investigated.

## 5 CONCLUSION

This study highlights CS training practices globally, showing substantial differences in between HIC and LMIC. These are particularly notable in the progression to independent surgical practice, competency assessment, and feedback approaches. The outcomes associated with CS are influenced by multiple patient‐ and system‐level factors, including access to care, patient risk profiles, and resources. However, training remains an essential and modifiable component, which, according to participants in this study, could be strengthened by incorporating evidence‐based educational practices such as structured training programs, including simulation‐based education, standardized procedures, and competency assessments. Future efforts should also foster collaboration between HIC and LMIC to improve maternal safety during CS. All mentors and trainers have a fundamental ethical and moral responsibility to deliver the highest quality training possible within their existing resource constraints.

## AUTHOR CONTRIBUTIONS

LDW and SG conceptualized the study, developed the survey, analyzed the results, and wrote the findings. EA and KC assisted with conceptualizing the article, analyzing the results, and writing the article. CJBM assisted with data analysis and interpretation.

## FUNDING INFORMATION

LW is supported by a Discovery Foundation Academic Fellowship Award.

## CONFLICT OF INTEREST STATEMENT

The authors of this paper have no conflict of interest to declare.

## ETHICS STATEMENT

The research proposal was granted approval by the Health Research and Ethics Committee of Stellenbosch University (S22/11/263) as a component of a larger study focusing on clinical skills training in April 2023.

## Supporting information


Data S1.

## Data Availability

The data that support the findings of this study are available from the corresponding author upon reasonable request.
