# Supplementary material for: Current practices in caesarean section training: A cross‐sectional study comparing high‐ and low‐middle‐income countries
Source: Int J Gynaecol Obstet. 2025 Dec 3;173(2):1012–25. doi: 10.1002/ijgo.70696 (PMC13094679; doi:10.1002/ijgo.70696)
Supplement: Supplementary file 1 — Data S1. [file IJGO-173-1012-s001.pdf]

Caesarean Section Training Survey

We would like to invite you to take part in a research project which involves the completion of an online questionnaire. The questionnaire should take you between 10-15 minutes to complete. Your participation is entirely voluntary and you are free to decline to participate or to stop completing the questionnaire at any time, even if you have agreed to take part initially. However, once you have submitted your completed questionnaire online, you will no longer be able to withdraw your responses as there will be no way of linking your responses back to you.

What is the study about?There is little data on educational practices in Caesarean Section (CS) training in especially Low-Middle income countries. In the scoping review of Zentner and colleagues on CS training, there were only 28 studies found, 17 (60%) set in High-income countries, 11 (40%) in Low and Middle-income countries.

This study aims to investigate current practices in Caesarean Section Training. This survey will be distributed through international societies in Obstetrics and Gynaecology, to involve as many participants as possible.

You are being asked to participate because you are a medical doctor performing or currently learning how to perform caesarean sections, and possibly teaching others to perform caesarean section.

If you agree to participate you will be requested to answer questions on how you were taught to perform caesarean sections, including details on how many and at which stage in your career. We will not ask you any personal information, this survey is completed anonymously.

What are the potential benefits of this research?To review methods used globally for teaching caesarean sections.To compare different strategies applied in Caesarean Section teaching around the world.To expand the knowledge and insight in the teaching methods for Caesarean Sections

Are there any risks involved in your taking part in this research?Participation in this survey will be anonymous, and no personal information will be collected.The information gathered will be factual and there are no emotional or physical risks anticipated.Collected information will be securely stored and password protected.

Permission to have all anonymous data shared with journals:When this study has been completed, the researcher(s) would like to publish results of the study in journals. Most journals require us to share your anonymous data with them before they publish the results. Therefore, we would like to obtain your permission to have your anonymous data shared with journals.

You can email the Principal Investigator of this study, Liesl de Waard at ldewaard@sun.ac.za if you have any questions about this study or encounter any problems.

This study has been approved by the Health Research Ethics Committee at Stellenbosch University (ID S22/11/263 PhD). The study will be conducted according to the ethical guidelines and principles of the international Declaration of Helsinki, and the Department of Health Ethics in Health Research: Principles, Processes and Studies (2015).

You can phone the Health Research Ethics Committee at +2721 938 9677/9819 if there still is something that concerns you about how this study is being conducted, or if you have a complaint.

By clicking AGREE, you are confirming that you:
- are over 18 years old;
- have read and understood the above explanation about the study;
- understand that your participation in this study is strictly voluntary; and
- you agree to participate

◯ AGREE

Demographics

1 What is your age?

____

2 Which gender identity do you identify with most?

◯ Female
◯ Male
◯ Non-binary
◯ Prefer to self-describe below (see follow-up question)
◯ Prefer not to say

2b You indicated you prefer to self-describe your gender identity. Please do so below:

____

3 What year did you complete your pre-graduate medical training (your primary medical degree)?

____

6 In what country did you complete your pre-graduate training (your primary medical degree)?

◯ Afghanistan
◯ Albania
◯ Algeria
◯ Andorra
◯ Angola
◯ Antigua and Barbuda
◯ Argentina
◯ Armenia
◯ Australia
◯ Austria
◯ Azerbaijan
◯ Bahamas
◯ Bahrain
◯ Bangladesh
◯ Barbados
◯ Belarus
◯ Belgium
◯ Belize
◯ Benin
◯ Bhutan
◯ Bolivia
◯ Bosnia and Herzegovina
◯ Botswana
◯ Brazil
◯ Brunei
◯ Bulgaria
◯ Burkina Faso
◯ Burundi
◯ Cabo Verde
◯ Cambodia
◯ Cameroon
◯ Canada
◯ Central African Republic
◯ Chad
◯ Chile
◯ China
◯ Colombia
◯ Comoros
◯ Congo
◯ Costa Rica
◯ Côte D'Ivoire
◯ Croatia
◯ Cuba
◯ Cyprus
◯ Czechia
◯ Democratic People's Republic of Korea
◯ Democratic Republic of the Congo
◯ Denmark
◯ Djibouti
◯ Dominica
◯ Dominican Republic
◯ Ecuador
◯ Egypt
◯ El Salvador
◯ Equatorial Guinea
◯ Eritrea
◯ Estonia
◯ Eswatini
◯ Ethiopia
◯ Fiji
◯ Finland
◯ France
◯ Gabon
◯ Gambia
◯ Georgia
◯ Germany
◯ Ghana

◯ Greece
◯ Grenada
◯ Guatemala
◯ Guinea
◯ Guinea-Bissau
◯ Guyana
◯ Haiti
◯ Honduras
◯ Hungary
◯ Iceland
◯ India
◯ Indonesia
◯ Iran
◯ Iraq
◯ Ireland
◯ Israel
◯ Italy
◯ Jamaica
◯ Japan
◯ Jordan
◯ Kazakhstan
◯ Kenya
◯ Kiribati
◯ Kuwait
◯ Kyrgyzstan
◯ Laos
◯ Latvia
◯ Lebanon
◯ Lesotho
◯ Liberia
◯ Libya
◯ Liechtenstein
◯ Lithuania
◯ Luxembourg
◯ Madagascar
◯ Malawi
◯ Malaysia
◯ Maldives
◯ Mali
◯ Malta
◯ Marshall Islands
◯ Mauritania
◯ Mauritius
◯ Mexico
◯ Micronesia
◯ Moldova
◯ Monaco
◯ Mongolia
◯ Montenegro
◯ Morocco
◯ Mozambique
◯ Myanmar
◯ Namibia
◯ Nauru
◯ Nepal
◯ Netherlands
◯ New Zealand
◯ Nicaragua
◯ Niger
◯ Nigeria
◯ North Macedonia
◯ Norway
◯ Oman
◯ Pakistan
◯ Palau
◯ Panama
◯ Papua New Guinea
◯ Paraguay
◯ Peru
◯ Philippines
◯ Poland

◯ Portugal
◯ Qatar
◯ Republic of Korea
◯ Romania
◯ Russian Federation
◯ Rwanda
◯ Saint Kitts and Nevis
◯ Saint Lucia
◯ Saint Vincent and the Grenadines
◯ Samoa
◯ San Marino
◯ Sao Tome and Principe
◯ Saudi Arabia
◯ Senegal
◯ Serbia
◯ Seychelles
◯ Sierra Leone
◯ Singapore
◯ Slovakia
◯ Slovenia
◯ Solomon Islands
◯ Somalia
◯ South Africa
◯ South Sudan
◯ Spain
◯ Sri Lanka
◯ Sudan
◯ Suriname
◯ Sweden
◯ Switzerland
◯ Syria
◯ Tajikistan
◯ Tanzania
◯ Thailand
◯ Timor-Leste
◯ Togo
◯ Tonga
◯ Trinidad and Tobago
◯ Tunisia
◯ Turkey
◯ Turkmenistan
◯ Tuvalu
◯ Uganda
◯ Ukraine
◯ United Arab Emirates
◯ United Kingdom
◯ United States
◯ Uruguay
◯ Uzbekistan
◯ Vanuatu
◯ Venezuela
◯ Vietnam
◯ Yemen
◯ Zambia
◯ Zimbabwe

---

8 In the country you trained in, what work do you do immediately after your primary medical degree?

◯ In your first post-graduate year you have an internship (work supervised in different disciplines)
◯ Work supervised as an intern in a chosen discipline
◯ You start specialising immediately after your medical degree
◯ Other

8b Please specify "other":

____

6 In what country are you currently or most recently working?

○ Afghanistan
○ Albania
○ Algeria
○ Andorra
○ Angola
○ Antigua and Barbuda
○ Argentina
○ Armenia
○ Australia
○ Austria
○ Azerbaijan
○ Bahamas
○ Bahrain
○ Bangladesh
○ Barbados
○ Belarus
○ Belgium
○ Belize
○ Benin
○ Bhutan
○ Bolivia
○ Bosnia and Herzegovina
○ Botswana
○ Brazil
○ Brunei
○ Bulgaria
○ Burkina Faso
○ Burundi
○ Cabo Verde
○ Cambodia
○ Cameroon
○ Canada
○ Central African Republic
○ Chad
○ Chile
○ China
○ Colombia
○ Comoros
○ Congo
○ Costa Rica
○ Côte D'Ivoire
○ Croatia
○ Cuba
○ Cyprus
○ Czechia
○ Democratic People's Republic of Korea
○ Democratic Republic of the Congo
○ Denmark
○ Djibouti
○ Dominica
○ Dominican Republic
○ Ecuador
○ Egypt
○ El Salvador
○ Equatorial Guinea
○ Eritrea
○ Estonia
○ Eswatini
○ Ethiopia
○ Fiji
○ Finland
○ France
○ Gabon
○ Gambia
○ Georgia
○ Germany
○ Ghana

◯ Greece
◯ Grenada
◯ Guatemala
◯ Guinea
◯ Guinea-Bissau
◯ Guyana
◯ Haiti
◯ Honduras
◯ Hungary
◯ Iceland
◯ India
◯ Indonesia
◯ Iran
◯ Iraq
◯ Ireland
◯ Israel
◯ Italy
◯ Jamaica
◯ Japan
◯ Jordan
◯ Kazakhstan
◯ Kenya
◯ Kiribati
◯ Kuwait
◯ Kyrgyzstan
◯ Laos
◯ Latvia
◯ Lebanon
◯ Lesotho
◯ Liberia
◯ Libya
◯ Liechtenstein
◯ Lithuania
◯ Luxembourg
◯ Madagascar
◯ Malawi
◯ Malaysia
◯ Maldives
◯ Mali
◯ Malta
◯ Marshall Islands
◯ Mauritania
◯ Mauritius
◯ Mexico
◯ Micronesia
◯ Moldova
◯ Monaco
◯ Mongolia
◯ Montenegro
◯ Morocco
◯ Mozambique
◯ Myanmar
◯ Namibia
◯ Nauru
◯ Nepal
◯ Netherlands
◯ New Zealand
◯ Nicaragua
◯ Niger
◯ Nigeria
◯ North Macedonia
◯ Norway
◯ Oman
◯ Pakistan
◯ Palau
◯ Panama
◯ Papua New Guinea
◯ Paraguay
◯ Peru
◯ Philippines
◯ Poland

◯ Portugal
◯ Qatar
◯ Republic of Korea
◯ Romania
◯ Russian Federation
◯ Rwanda
◯ Saint Kitts and Nevis
◯ Saint Lucia
◯ Saint Vincent and the Grenadines
◯ Samoa
◯ San Marino
◯ Sao Tome and Principe
◯ Saudi Arabia
◯ Senegal
◯ Serbia
◯ Seychelles
◯ Sierra Leone
◯ Singapore
◯ Slovakia
◯ Slovenia
◯ Solomon Islands
◯ Somalia
◯ South Africa
◯ South Sudan
◯ Spain
◯ Sri Lanka
◯ Sudan
◯ Suriname
◯ Sweden
◯ Switzerland
◯ Syria
◯ Tajikistan
◯ Tanzania
◯ Thailand
◯ Timor-Leste
◯ Togo
◯ Tonga
◯ Trinidad and Tobago
◯ Tunisia
◯ Turkey
◯ Turkmenistan
◯ Tuvalu
◯ Uganda
◯ Ukraine
◯ United Arab Emirates
◯ United Kingdom
◯ United States
◯ Uruguay
◯ Uzbekistan
◯ Vanuatu
◯ Venezuela
◯ Vietnam
◯ Yemen
◯ Zambia
◯ Zimbabwe

5 What is your current job title? (or if you are retired or not currently working your most resent job title)

◯ Intern (year-one post-graduate training, not specializing, working under supervision)
◯ Medical officer (post-graduate non-specialist doctor)
◯ Registrar/trainee specializing in obstetrics and gynaecology
◯ Registrar/trainee specializing in another discipline
◯ Specialist, obstetrics and gynaecology
◯ Specialist, other discipline
◯ Other

5b Please specify "other":

____

4 How many years have you worked in an obstetric service where you are or were involved with performing Caesarean Sections?

◯ 0-5 years
◯ 6-10 years
◯ 11-15 years
◯ 16-20 years
◯ 21-25 years
◯ 26-30 years
◯ >30 years

7 In the country you are working in who primarily performs Caesarean Sections?

◯ Medical specialists - Obstetrics and Gynaecology
◯ Other specialists
◯ Non-specialist doctors who have completed their training
◯ Doctors in training as specialist
◯ Other

7b Please specify "other":

____

Caesarean Section Experience

6 What country did you train to perform Caesarean Sections in?

◯ Afghanistan
◯ Albania
◯ Algeria
◯ Andorra
◯ Angola
◯ Antigua and Barbuda
◯ Argentina
◯ Armenia
◯ Australia
◯ Austria
◯ Azerbaijan
◯ Bahamas
◯ Bahrain
◯ Bangladesh
◯ Barbados
◯ Belarus
◯ Belgium
◯ Belize
◯ Benin
◯ Bhutan
◯ Bolivia
◯ Bosnia and Herzegovina
◯ Botswana
◯ Brazil
◯ Brunei
◯ Bulgaria
◯ Burkina Faso
◯ Burundi
◯ Cabo Verde
◯ Cambodia
◯ Cameroon
◯ Canada
◯ Central African Republic
◯ Chad
◯ Chile
◯ China
◯ Colombia
◯ Comoros
◯ Congo
◯ Costa Rica
◯ Côte D'Ivoire
◯ Croatia
◯ Cuba
◯ Cyprus
◯ Czechia
◯ Democratic People's Republic of Korea
◯ Democratic Republic of the Congo
◯ Denmark
◯ Djibouti
◯ Dominica
◯ Dominican Republic
◯ Ecuador
◯ Egypt
◯ El Salvador
◯ Equatorial Guinea
◯ Eritrea
◯ Estonia
◯ Eswatini
◯ Ethiopia
◯ Fiji
◯ Finland
◯ France
◯ Gabon
◯ Gambia
◯ Georgia
◯ Germany
◯ Ghana

◯ Greece
◯ Grenada
◯ Guatemala
◯ Guinea
◯ Guinea-Bissau
◯ Guyana
◯ Haiti
◯ Honduras
◯ Hungary
◯ Iceland
◯ India
◯ Indonesia
◯ Iran
◯ Iraq
◯ Ireland
◯ Israel
◯ Italy
◯ Jamaica
◯ Japan
◯ Jordan
◯ Kazakhstan
◯ Kenya
◯ Kiribati
◯ Kuwait
◯ Kyrgyzstan
◯ Laos
◯ Latvia
◯ Lebanon
◯ Lesotho
◯ Liberia
◯ Libya
◯ Liechtenstein
◯ Lithuania
◯ Luxembourg
◯ Madagascar
◯ Malawi
◯ Malaysia
◯ Maldives
◯ Mali
◯ Malta
◯ Marshall Islands
◯ Mauritania
◯ Mauritius
◯ Mexico
◯ Micronesia
◯ Moldova
◯ Monaco
◯ Mongolia
◯ Montenegro
◯ Morocco
◯ Mozambique
◯ Myanmar
◯ Namibia
◯ Nauru
◯ Nepal
◯ Netherlands
◯ New Zealand
◯ Nicaragua
◯ Niger
◯ Nigeria
◯ North Macedonia
◯ Norway
◯ Oman
◯ Pakistan
◯ Palau
◯ Panama
◯ Papua New Guinea
◯ Paraguay
◯ Peru
◯ Philippines
◯ Poland

◯ Portugal
◯ Qatar
◯ Republic of Korea
◯ Romania
◯ Russian Federation
◯ Rwanda
◯ Saint Kitts and Nevis
◯ Saint Lucia
◯ Saint Vincent and the Grenadines
◯ Samoa
◯ San Marino
◯ Sao Tome and Principe
◯ Saudi Arabia
◯ Senegal
◯ Serbia
◯ Seychelles
◯ Sierra Leone
◯ Singapore
◯ Slovakia
◯ Slovenia
◯ Solomon Islands
◯ Somalia
◯ South Africa
◯ South Sudan
◯ Spain
◯ Sri Lanka
◯ Sudan
◯ Suriname
◯ Sweden
◯ Switzerland
◯ Syria
◯ Tajikistan
◯ Tanzania
◯ Thailand
◯ Timor-Leste
◯ Togo
◯ Tonga
◯ Trinidad and Tobago
◯ Tunisia
◯ Turkey
◯ Turkmenistan
◯ Tuvalu
◯ Uganda
◯ Ukraine
◯ United Arab Emirates
◯ United Kingdom
◯ United States
◯ Uruguay
◯ Uzbekistan
◯ Vanuatu
◯ Venezuela
◯ Vietnam
◯ Yemen
◯ Zambia
◯ Zimbabwe

---

9 Are you currently performing Caesarean Sections?

◯ Yes, independently
◯ Yes, under supervision
◯ No, I am in early training
◯ Other

9b Please specify "other":

____

10 What year did you start performing Caesarean Sections?

____

Approximately how many caesarean sections have you performed independently?

____

Caesarean Section Training

11 How many years after completing your pre-graduate medical degree did you start with Caesarean Section training?

____

12 Did you receive any training for Caesarean Sections during your primary medical (undergraduate) degree? (select all that apply)

- ☐ No
- ☐ Yes, lectures only
- ☐ Yes, observation only
- ☐ Yes, skill simulation / in skills lab
- ☐ Yes, in the operating room assisting
- ☐ Yes, in the operating room performing certain aspects (e.g. closing the rectus sheath)
- ☐ Yes, in the operating room performing the complete procedure
- ☐ Other

12b Please specify "other":

____

13 Regarding your training in Caesarean Sections, was there a formal curriculum/training program used for the Caesarean Section training? (choose all that apply)

- ☐ No
- ☐ Yes during my primary medical degree
- ☐ Yes after my primary medical degree
- ☐ Yes during my specialty training
- ☐ Unsure

14 Which of the following methods were used for your Caesarean Section training? (select all that apply)

- ☐ Apprenticeship model (assist until trainer deems you competent to perform the Caesarean Section)
- ☐ Simulation training (simulators / models used to teach steps of a Caesarean Section)
- ☐ Pre-surgical training courses/modules
- ☐ Observation only
- ☐ None
- ☐ Other

14b Please specify "other"

15 How is feedback given during training? (select all that apply)

- ☐ Written
- ☐ Formal verbal feedback (i.e. trainer and trainee have a planned discussion regarding the details of the surgical progress)
- ☐ Informal verbal feedback during and after cases (comments on technique and progress)
- ☐ Correcting techniques or steps during the procedure, for the sake of patient safety
- ☐ None / I am not aware of any feedback being given
- ☐ Other

15b Please specify "other"

16 Approximately how many cases did you assist with before you started performing parts of a Caesarean Section on your own?

____

17 Approximately how long did you assist before you started performing parts of a Caesarean Section on your own?

◯ < 1 month
◯ 1-6 months
◯ 0.5-1 year
◯ 1-5 years
◯ >5 years

18 Approximately how many cases did you perform partially before your first entire Caesarean Section case?

____

19 Approximately how many cases did you perform supervised before you performed Caesarean Sections independently?

____

20 At what stage in your career did you perform Caesarean Sections independently? (assisted by someone at a lower level of experience than yourself)

◯ Intern (year one post-graduate training, not specialising, working under supervision)
◯ Medical officer (post-graduate but non-specialist doctor)
◯ Registrar/trainee specialising in obstetrics and gynaecology
◯ Registrar/trainee specialising in another discipline
◯ Specialist, obstetrics and gynaecology
◯ Specialist, other discipline
◯ Other

20b Please specify "other"

____

21 Approximately how long did you perform Caesarean Sections under supervision before operating independently?

◯ < 1 month
◯ 1-6 months
◯ 0.5-1 year
◯ 1-5 years
◯ >5 years

22 Was there any formal process to determine your competence prior to you performing a Caesarean Section independently? (select all that apply)

☐ No, there was no formal process to determine my competence
☐ Yes, informal assessment by supervisors
☐ Yes, formal structured assessment by supervisor
☐ Yes, logbook or portfolio assessment
☐ Other

22b Please specify "other"

Experience of Performing Caesarean Section

23 When you performed your first Caesarean Section independently, reflecting back, how adequate was your training?

- ◯ Extremely inadequate
- ◯ Inadequate
- ◯ Somewhat inadequate
- ◯ Neutral
- ◯ Somewhat adequate
- ◯ Adequate
- ◯ Extremely adequate

24 When you performed your first Caesarean Section independently, how anxious did you feel on a scale of 1-7?

- ◯ 1 - Not anxious at all
- ◯ 2
- ◯ 3
- ◯ 4
- ◯ 5
- ◯ 6
- ◯ 7 - Extremely anxious

25 When you performed your first Caesarean Section independently, how confident did you feel on a scale of 1-7?

- ◯ 1 - Not confident at all
- ◯ 2
- ◯ 3
- ◯ 4
- ◯ 5
- ◯ 6
- ◯ 7 - Extremely confident

26 When you performed your first Caesarean Section independently, how excited did you feel on a scale of 1-7?

- ◯ 1 - Not excited at all
- ◯ 2
- ◯ 3
- ◯ 4
- ◯ 5
- ◯ 6
- ◯ 7 - Extremely excited

27 Did you experience complications during your first year of performing Caesarean Sections independently?

- ◯ No, I did not experience complications that I am aware of
- ◯ Yes, I experienced minor surgical complications requiring correction by a senior colleague during the procedure
- ◯ Yes, I experienced major surgical complications requiring additional surgical procedures such as a relook laparotomy
- ◯ Yes, I experienced surgical-associated maternal mortality
- ◯ Yes, I experienced surgical complications and surgical-associated maternal mortality

28 How satisfied are you with the Caesarean Section training you received?

◯ Extremely unsatisfied
◯ Unsatisfied
◯ Somewhat unsatisfied
◯ Neutral
◯ Somewhat satisfied
◯ Satisfied
◯ Extremely satisfied

29 How do you think Caesarean Section training could be improved?

30 What do you think is good about Caesarean Section training as it is now?

Caesarean Section Teaching

31 Are you currently training others in performing Caesarean Section?

◯ No
◯ Not now, but I have in the past
◯ Yes

The rest of this section is for respondents who train others in performing Caesarean Sections. You may now skip to the following section (question 36).

32 Do you use any formal training program/curriculum while training others?

◯ No
◯ Yes
◯ Unsure

32b Please specify the training program/curriculum you use:

33 Which of the following methods do you use for Caesarean Section training? (select all that apply)

☐ Apprenticeship model (assist until trainer deems you competent to perform the Caesarean Section)
☐ Simulation training - simulators/models used to teach steps of Caesarean Section
☐ Pre-surgical training (e.g. lectures, tutorials, online content)
☐ Observation only
☐ None
☐ Other

33b Please specify "other":

____

33c You indicated you use pre-surgical training as a teaching method. Which of the following methods do you use? (select all that apply)

☐ Lectures
☐ Tutorials
☐ Online content
☐ Other

33d Please specify which other pre-surgical training methods you use:

____

34 How is feedback given during the Caesarean Section training you provide? (select all that apply)

☐ Written
☐ Formal verbal feedback (i.e. the trainer and trainee have a planned discussion regarding the details of the surgical progress)
☐ Informal verbal feedback during and after cases (comments on technique and progress)
☐ Correcting techniques or steps during the procedure, for the sake of patient safety
☐ None / I am unaware of feedback being given
☐ Other

34b Please specify "other":

____

35 Do you think there is a need for a formal training program for Caesarean Sections?

◯ No
◯ Yes
◯ Unsure

35b You indicated you believe there is a need for a formal training program for Caesarean Sections. What kind of program would you suggest?

35c What do you believe would be the expected benefits of having a formal training program for Caesarean Sections?

35d You indicated you believe there is no need for a formal training program for Caesarean Sections. Please explain why not:

End of Survey

36 You have reached the end of this survey. Do you have any comments for the research team?

◯ No
◯ Yes

36b Please leave your comments below:
